# Microspheres as a Carrier System for Therapeutic Embolization Procedures: Achievements and Advances

**DOI:** 10.3390/jcm12030918

**Published:** 2023-01-24

**Authors:** Mick. M. Welling, Nikolas Duszenko, Maarten P. van Meerbeek, Tom J. M. Molenaar, Tessa Buckle, Fijs W. B. van Leeuwen, Daphne D. D. Rietbergen

**Affiliations:** 1 Interventional Molecular Imaging Laboratory, Department of Radiology, Leiden University Medical Center, 2333 ZA Leiden, The Netherlands; 2 Departments of Parasitology and Infectious Diseases, Leiden University Medical Center, 2333 ZA Leiden, The Netherlands; 3 Radiochemistry Facility, Department of Radiology, Leiden University Medical Center, 2333 ZA Leiden, The Netherlands; 4 Section of Nuclear Medicine, Department of Radiology, Leiden University Medical Center, 2333 ZA Leiden, The Netherlands

**Keywords:** microspheres, embolization, hepatocellular carcinoma, therapy, TAE, TACE, TARE

## Abstract

The targeted delivery of anti-cancer drugs and isotopes is one of the most pursued goals in anti-cancer therapy. One of the prime examples of such an application is the intra-arterial injection of microspheres containing cytostatic drugs or radioisotopes during hepatic embolization procedures. Therapy based on the application of microspheres revolves around vascular occlusion, complemented with local therapy in the form of trans-arterial chemoembolization (TACE) or radioembolization (TARE). The broadest implementation of these embolization strategies currently lies within the treatment of untreatable hepatocellular cancer (HCC) and metastatic colorectal cancer. This review aims to describe the state-of-the-art TACE and TARE technologies investigated in the clinical setting for HCC and addresses current trials and new developments. In addition, chemical properties and advancements in microsphere carrier systems are evaluated, and possible improvements in embolization therapy based on the modification of and functionalization with therapeutical loads are explored.

## 1. Introduction

The targeted delivery of anti-cancer drugs and isotopes is the most pursued goal in anti-cancer therapy. However, a major disadvantage of the systemic application of these drugs is their poor selectivity for cancer cells and their general distribution to non-cancerous tissues, causing toxic side effects to otherwise healthy tissue [1,2]. To this end, novel therapies are constantly being discovered and applied for anti-cancer interventions in pursuit of avoiding toxic side effects. Theoretically, the involvement of non-cancerous tissues can also be reduced via disease-targeted therapeutic delivery. Herein, disease-targeting can be guided by either receptor targeting or via selective administration [3]. Unique for the hepatic system is that cancerous diseases such as HCC and mCRC alter the vascularization of the liver. Normal liver tissue will receive oxygen from the portal system; HCC and mCRC receive oxygen from the artery and are therefore suitable for trans-arterial therapy [4]. Over the past two decades, microspheres have proven themselves effective trans-arterial drug delivery systems [5]. Such microspheres have broad applications in both life science research and medicine [6], e.g., contrast agents [7], tissue fillers [8], and drug delivery vehicles [9]. Stable or biodegradable microspheres are generally utilized to direct drugs to organs by taking advantage of vascular physical restraints [10,11,12,13,14]. Therapeutic microspheres are characteristically small particles or beads with a well-defined size distribution of 50–750 mm, consisting of either glass, synthetic polymers, or proteins [15]. By selectively blocking the blood supply of the targeted tissue via so-called trans-arterial embolization (TAE), the deprived tissue will be starved of nutrients, ultimately resulting in a therapeutic benefit. When lodged in the end vasculature that surrounds the tumor, microspheres can also release encapsulated drugs (trans-arterial chemoembolization (TACE)) or irradiate surrounding tissue via embedded radioisotopes (trans-arterial radioembolization (TARE)).

Being a medicinal product, microspheres are considered medical devices rather than drugs. From a practical perspective, this means that such agents have to comply with different regulations compared to more standard therapeutics. Independent of this classification, the various clinical requirements ultimately drive therapeutical applications, of which the most important ones are stated in Table 1. Caine et al. extensively reviewed more detailed information on the microspheres used for TAE [16].

Therapeutical microspheres have been widely implemented for hepatocellular cancer (HCC) and hepatic metastasized disease of different kinds of solid cancers, including colorectal, lung, and breast cancer that metastasize to the liver [5,17]. Non-treatable advanced neoplastic diseases and the development of hepatic metastasis have poor prognoses. Only 10–20% of these patients are suitable candidates for radical resection, as surgical excision cannot be applied in grade 3–5 staged HCC [18]. A substantial portion of inoperable patients who present metastatic liver tumors needs alternative treatment strategies or therapy for bridging surgery [19,20]. Alternative therapies encompass minimally invasive techniques, including percutaneous ablative treatments (radiofrequency ablation, microwave ablation) and trans-catheter intra-arterial therapies [21,22]. 

**Scheme 1:**
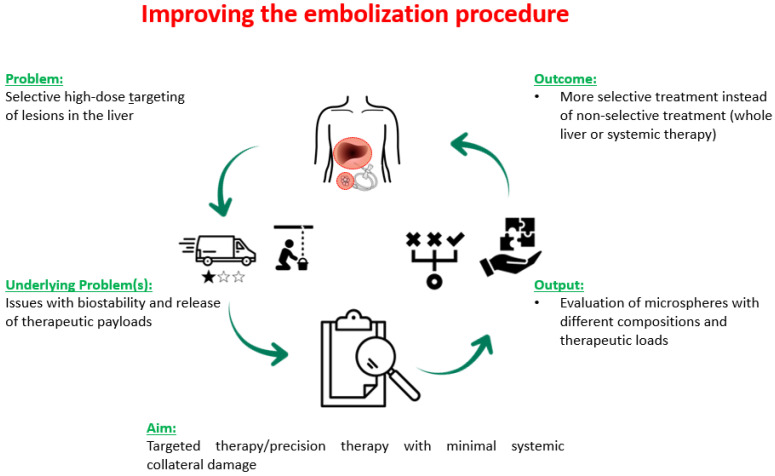
Theory of change for improving therapeutic embolization procedures using microspheres in HCC.

With a focus on HCC, this review provides an overview of the properties and applications of various microspheres and microsphere carrier systems’ chemical properties and advancements. In addition, the latest developments in embolization strategies and alternative technologies are discussed (Scheme 1). 

## 2. Trans-Arterial Embolization for Vascular Occlusion

**Figure 1:**
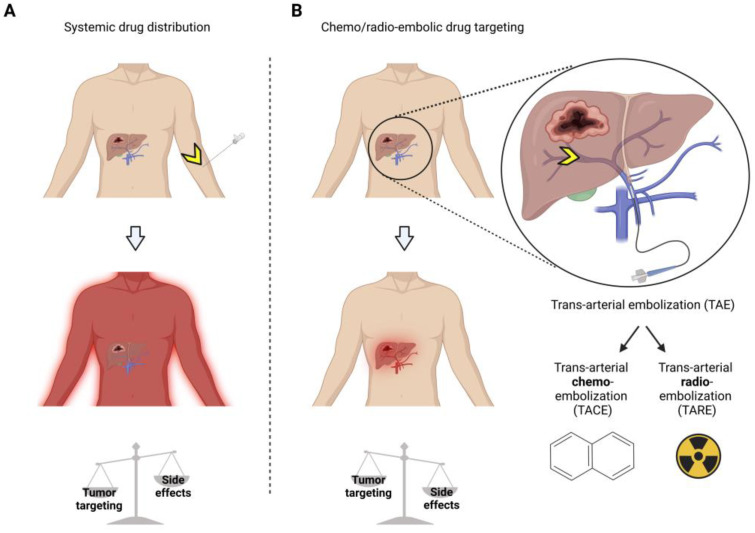
Treatment of hepatocellular carcinoma (HCC) shows the advantages of chemo/radio-embolization drug targeting over systemic drug therapy. Systemic drug therapy (**A**) yields lower tumor targeting and more side effects than local delivery via trans-arterial chemo/radio embolic drug targeting (**B**). Yellow arrows = injection site. Created with BioRender.com.

Trans-arterial embolization (TAE) is a technique wherein inert microspheres are used to block the blood supply around the tumor. The use of large microspheres, e.g., Embosphere^®^ (⌀ 300–750 mm, Merit Medical Systems, South Jordan, UT, USA) in tumor-affected liver lobe(s) allows blockage of the blood supply towards the tumor (Figure 1B), ultimately resulting in reduced tumor growth [10,11,12,13,14]. An overview of the different microspheres currently used for hepatic trans-arterial embolization is provided in Table 2. The clinically applied glass and synthetic spheres are non-degradable and remain in the vasculature for life. Recently, in a pre-clinical phase, biodegradable spheres were developed, which allow local occlusion of the vasculature, and after degradation they allow a follow-up injection of embolization treatment. The clinical unfavorable short- and long-term outcomes of patients with large HCCs (≥50 mm) were revealed compared to those with small HCCs (<50 mm). Detailed analyses revealed that the average rates of change in tumor size and shrinkage after TAE were 48.6 ± 35.6 mm and 30.7 ± 17.0%, respectively [23]. Additional details on the chemical properties and advancements of microsphere carrier systems are evaluated in Section 4.

Instead of focusing on major vascular occlusion, chemo- or radio-embolic targeting is an alternative treatment option with microspheres that have the potential to manage hepatic tumors effectively [18].

## 3. Therapeutic Loads Employed during Microsphere-Trans-Arterial Embolization Therapy

### 3.1. Trans-Arterial Chemoembolization

Trans-arterial chemoembolization (TACE) uses embolization to deliver chemotherapy locally, thus limiting systemic exposure (Figure 1) [30,31]. One prime example are acrylic co-polymer microspheres (Hepasphere™, ⌀ 50–100 µm, Merit Medical Systems, Inc., South Jordan, UT, USA) that can absorb cytostatic drugs such as doxorubicin, irinotecan, epirubicin, mitomycin, cisplatin, and oxaliplatin (Table 3). 

After contact with either an ionized environment, such as 0.9% NaCl and blood, or nonionic contrast media, acrylic co-polymer microspheres expand to 200–400 µm and slowly release their cytostatic payload [50]. The advantage of TACE is that it maximizes the concentration of chemotherapeutic agents within the tumor for up to seven days while keeping a minimal concentration in the systemic circulation. This approach reduces systemic side effects and the toxicity of cytostatic drugs compared to systemic chemotherapy. Furthermore, the malignancy’s arterial supply is occluded like in TAE, thus limiting nutrient availability to the tumor [51,52]. In HCC patients [5], this chemoembolization strategy has proven beneficial for patients’ survival, increasing the survival time by up to 12 months [53] and reducing the symptoms related to chemotherapy [28].

### 3.2. Trans-Arterial Radioembolization (TARE)

Trans-arterial radioembolization (TARE) uses radioisotopes embedded in microspheres to locally irradiate tissue after the vascular occlusion of blood vessels surrounding the tumor [54]. Herein, microspheres carrying b-emitting radioisotopes enable a more pinpointed delivery of radiation to liver tumors than other radiotherapy techniques. Although these options extend patient survival, most remain palliative [10,53]. With ^166^Ho-microspheres and 3.8 GBq/kg liver tissue in a Phase II study including 38 patients, the target lesions showed complete response or stabilized disease for 27 patients (73%), with a median survival of 15 months [55]. Given these results, a more extensive, randomized Phase III study appears to be required. Although ^90^Y, ^188^Re, and ^166^Ho in microspheres effectively reduce tumor size and patients’ survival, data from large Phase III trials are warranted to prove their benefits compared to other treatment modalities. In addition, the cost-effectiveness between the various radioisotopes and types of microspheres has to be determined. Besides the application of TARE in HCC, patients with intrahepatic cholangiocarcinoma (CC), which is a rare but very aggressive neoplasia with limited therapeutic options, and patients with to liver metastasized colorectal (mCRC) and neuroendocrine disease also benefit from therapy using ^90^Y-loaded glass or resin microspheres, with a response of more prolonged overall survival of at least 6 months [56,57,58,59]. Various radioembolization microspheres carrying radioisotopes to be delivered in the tumor-bearing hepatic segments of patients are summarized in Table 4. This table is specified in non-degradable and degradable microspheres. As we noted for TAE and TACE, the non-degradable microspheres allow only one treatment session due to the permanent occlusion of the vasculature. In pre-clinical settings, biodegradable microspheres have been evaluated for their effectiveness in TARE, as described in Section 4.

An essential issue in radioembolization studies is preventing shunting to normal tissues, such as the lungs. Shunting displaces a fraction of the administered particles towards the microvasculature of other tissues, mainly the lung, instead of the liver, leading to ineffective dose distribution and irreversible severe adverse effects such as radiation pneumonitis [88,94]. To avoid the shunting of microspheres, radioembolization is performed in a theranostic setting. In this setting, a catheter is (selectively) placed to deliver radiolabeled macro-aggregated albumin (^99m^Tc-MAA; ⌀ 10–40 mm) to the affected tissues. Via a scout scan using single-positron emission computed tomography (SPECT) imaging, the localization and distribution of the ^99m^Tc-MAA are visualized, which is an approach that helps assess the degree of shunting and, at the same time, facilitates dosimetry measurements. When this has been done, therapeutic loads of β-emitting glass or resin microparticles (⌀ 15–25 mm) [95] containing, e.g., ^90^Y (SIR-Spheres^®^, Sirtex Medical Inc. Woburn, MA, USA; Therasphere, Boston Scientific, Marlborough, MA, USA) or ^166^Ho (QuiremSpheres, Quirem Medical Deventer, The Netherlands) are injected via a catheter positioned in the same way [96]. Given the overlap in size and retention properties between ^99m^Tc-MAA and microparticles, a ^99m^Tc-MAA scout scan has been deemed a sufficient standard requirement in the clinical guidelines to predict the accurate delivery of therapeutic microspheres. Despite these guidelines, a mismatch between the scout and therapeutic is inevitable, i.e., given the time span that separates these two procedures. As such, the delivery of microspheres can still lead to adverse side effects and suboptimal dose delivery in about 30% of cases [97,98,99], a complication which highlights the need for innovative solutions that help refine the correlation between the scout scan and therapeutic delivery. In this respect, the physical properties of the used radioisotope, ^199^Ho as a b/g/paramagnetic element [100] and ^90^Y as a PET/SPECT imaging agent, post-TARE imaging facilitates mapping of the dose delivery. Where the therapy is insufficient, adjuvant therapy can be considered [101,102]. Furthermore, microspheres containing these isotopes can also serve as a scout scan. Another drawback is the delay of 2 weeks between the execution of the scout scan and the therapeutic intervention due to the need for dosimetry [103,104,105] and the production/delivery time of the β-emitting microspheres [96].

### 3.3. TACE vs. TARE

Instead of focusing on major vascular occlusion with TAE, in this section, we focus on comparing chemo- or radio-embolic targeting that has the potential to facilitate vascular occlusion and realize chemo- or radio-embolic treatment of hepatic tumors [18].

Four TARE studies determined the overall survival median at 9–11 months [50]. Based on these findings, TARE was not recommended as a first-line therapy for patients with non-resectable colorectal liver metastasis. For HCC, however, the overall survival in a study with unresectable HCC patients was 19.9 months in the ^90^Y-resin TARE group, which was an improvement compared to the 14 months of survival in a matching TACE group [106]. Recently, the efficacy of TARE combined with TACE was determined in 19 patients with bi-lobar HCC, and no procedure-related major clinical complications were observed, and the mean overall survival yielded a promising 27.3 months compared to untreated patients [107]. Differences between studies comparing TACE and TARE indicate that the outcome in the benefits of treatment may be related to the type of carcinoma, an observation that needs additional research. TARE also proved superior in safety regarding post-embolization syndrome, hospitalization days, and outpatient-based therapy [108,109]. TARE was a safe alternative treatment to TACE [110], especially as using a scout scan helps prevent complications related to shunting with TACE [111]. Applying the scout scan also helps personalize the dosing, a concept that could extend to TACE. Compared to TACE, TARE had a longer time-to-progression, greater ability to downsize tumors, and less post-embolization syndrome [112]. For that reason, it could be an alternative to ablation, surgical resection, or portal vein embolization [113]. On the other hand, TACE is the trans-arterial treatment of choice for patients with marginal hepatic reserve (i.e., hyperbilirubinemia, ascites) or candidates for transplantation [114]. 

## 4. Chemical Properties and Advancements of Microsphere Carrier Systems

**Figure 2:**
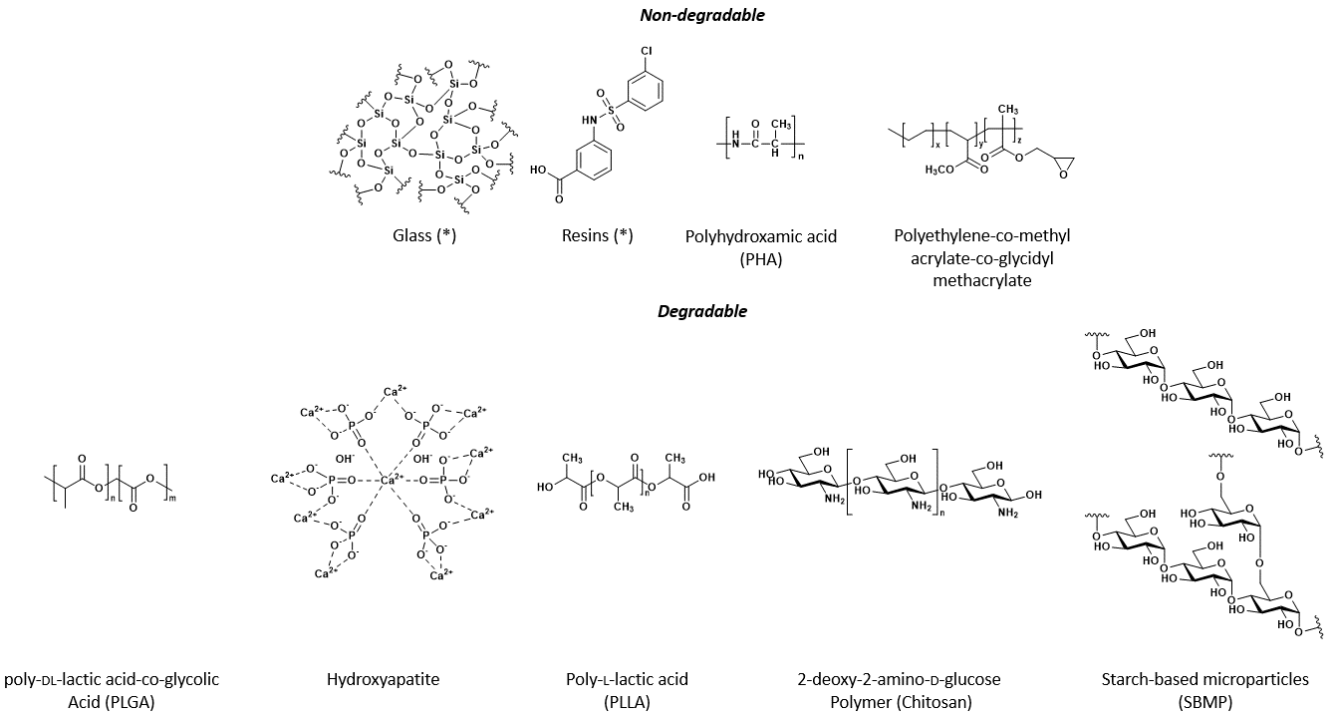
Schematic representations of building blocks and backbones for constructing radioactive microspheres. * = details binding unknown, insertion after mixing.

**Figure 3:**
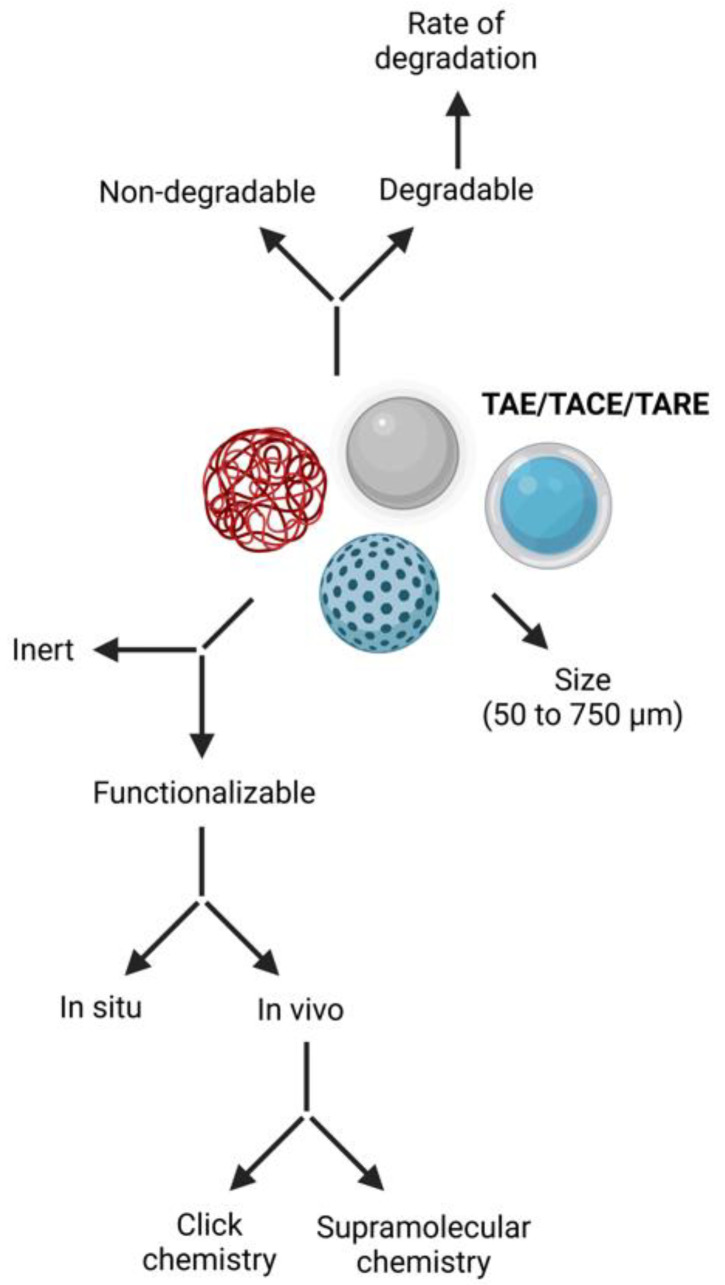
Overview of microsphere properties and functionalizations. Details regarding the clinical features and pre-clinical set-ups are described in Section 2, Section 3 and Section 4. Created with BioRender.com.

This section evaluates the chemical properties and advancements of radioactive functionalized microsphere carrier systems. A summary of these properties and modifications of microspheres is depicted in Figure 2 and Figure 3.

### 4.1. Non-Degradable Microspheres

Several examples are available for the use of non-degradable microspheres in embolization procedures. Such particles are generally chemically stable and can be sterilized and monodispersed with a very tight particle-size distribution. Therapy with non-degradable microspheres is only suitable for a single embolization intervention and remains in the vasculature indefinitely. Examples of such non-degradable particles are discussed below.

- Chemically inert glass—microspheres are non-porous, do not induce immunological effects, and are FDA-approved for application in humans for embolization therapy. Glass microspheres have a primary yttria–alumina–silica system (YAS), and a ternary YAS composition (40% Y_2_O_3_, 40% SiO_2_, and 20% Al_2_O_3_) [66]. These microspheres, on average, have a diameter of 50–150 mm. Glass is used in clinical TAE and TARE therapies [115,116]. Radioisotopes can be embedded during preparation into the glass via thermal neutron irradiation in a nuclear reactor (Figure 2). In this process, irradiation of stable Y—through an ^89^Y (*n*, γ)^90^Y reaction—produces ^90^Y [43,117]. The amount can be fine-tuned between 0.5 and 11 GBq per treatment according to need [118]. These glass microspheres cannot be used for TACE as elution of embedded drugs and surface modifications are impossible.
- Ion-exchange resin-based microspheres SIR-Spheres^®^ are porous, have a lower density/weight than glass, and are regularly used for TAE and TARE [54]. These polymers do not contain any groups amenable to covalent conjugation. Various resins were investigated for TARE only after including stable Y, Ho, or Sm, preparing microspheres during the synthesis process (Figure 2). As for glass microspheres, neutron-activated formulations in a research reactor after irradiation of stable Y, Ho, or SM isotopes through (*n*, γ) reaction procedures yielded ^90^Y, ^166^Ho, or ^153^Sm [43,54]. Bio-Rex 70 (Bio-Rad Inc. Veenendaal, The Netherlands) proved to have the best properties in stability, loading capacity, and sterilization [119]. Next to high-energy beta-radiation, ^166^Ho also emits gamma-radiation, which allows for imaging by gamma scintigraphy, thus helping determine treatment dose. ^153^Sm and ^166^Ho have a theragnostic advantage as they emit both therapeutic beta and diagnostic gamma radiations, allowing both imaging and therapy in one. In combination with ^153^Sm, this resin was pursued as an alternative microsphere in light of production time, stability, and costs [120,121].
- The discovery of the macroporous chelating ion exchanger G-Gel (Merck, Darmstad, Germany), consisting of poly(glycidyl methacrylate-co-ethylene dimethacrylate), helps provide a new class of ^177^Lu and ^131^I carriers [122]. Methacrylate is formed in beads that support high radionuclide loading due to macroporous structure and mechanically stable sphere-shaped particles of 20–40 μm. This concept is being implemented in G-Gel [43]. The material facilitates the conjugation of functional moieties, such as chelates and dyes, without negatively impacting the overall properties [70]. In this respect, DOTA and Quinoline-8-ol have been used as metal-binding ligands because they readily form stable complexes with nearly all therapeutically or diagnostically used metal ion radionuclides such as ^90^Y, ^188^Re, ^166^Ho, and ^177^Lu [54]. This stable complexation makes G-Gel less useful for drug release as with TACE.
- The interest in using cellulose for TARE comes from its nontoxicity, biocompatibility, biodegradability, and amenable chemistry for functionalization with, e.g., chelating groups. Polyhydroxyamic acid polyacrylamide (PHA) has been chosen for its capacity to form complexes with a wide range of metallic radionuclides [71,123]. In one study on the efficacy of a PHA loaded with ^177^Lu [123], PHA microspheres were synthesized starting from polyacrylamide. Incorporating isotopes such as ^177^Lu seems straightforward; modification of the polymers to incorporate dyes/chelates/adamantane seems impossible due to the complicated chemistry. Thus, PHA-functionalized microspheres were not applied for TACE, as is the case for resin microspheres. Subsequently, experimental variables such as reaction pH, amount of PHA microspheres, carrier ^177^Lu content, and incubation time were optimized for maximum uptake of ^177^Lu on PHA microspheres (median particle size to be 54 μm, which is still suitable for TARE, but relatively small for an effective TAE). Under optimized conditions, >99% loading of ^177^Lu on PHA microspheres with high stability could be achieved. ^177^Lu-PHA microspheres exhibited excellent in vitro stability in sodium phosphate solutions, saline, and serum for up to 5 days at 37 °C. In animal studies, 93% of ^177^Lu-PHA microspheres were retained in the liver at 96 h post-injection without significant leakage to other organs. Although the latter is encouraging, this set-up has not yet been evaluated in patients for HCC [71].

### 4.2. Bio-Degradable Particles

Biodegradable microspheres have the potential to provide an alternative to stable microspheres, and they can potentially allow sequential microsphere administrations. Repeated injections are an advantage, considering that a single treatment of HCC using TACE or TARE may not be sufficient for a successful remission. Moreover, they can be armed with bifunctional payloads, e.g., radioisotopes and elution of anti-tumor drugs. The following section summarizes various microspheres’ characteristics and applications (Table 3, Figure 2) and mentions the FDA clearance. For TAE only, biodegradable microspheres have not been evaluated.

- Poly-DL-lactic-co-glycolic acid (PLGA) particles can be formed to the size of microspheres by employing emulsion or microemulsion polymerization, interfacial polymerization, and precipitation polymerization, and a monomer as a starting point [124]. Thereafter, they can be modified into biodegradable carriers for the controlled delivery of drugs and isotopes. PLGA has one reactive COOH group per polymer chain. Functionalization of PLGA microspheres should be possible, although the influence on the hydrogel formation in combination with PEG is unclear. PLGA is widely used for TACE [44,45,46], although the process of drug release is complex [125]. In general, drug release occurs mainly via diffusion through pores, osmotic pumping, degradation, or erosion. More recently, modifications for TARE have been initiated [126]. An application of PLGA in TARE is in Radiogel^®^ (Vivos Inc., Richland, WA, USA), registered as a medical device under the FDA. InjecTable ^90^Y-Radiogel^®^ comprises an insoluble ^90^Y-phosphate (YPO_4_) radiation source mixed within an injectable, thermally reversible, temperature-sensitive polymer solution that includes polylactide, polyglycolide, and polylactic-co-glycolic acid co-polymers, all embedded in a microsphere [126]. This hydrogel is a liquid at temperatures below body temperature but begins to gel and harden upon injection as the temperature increases to normal body temperature, thereby locking the particles in place. RadioGel^®^ is drained within tumor extracellular spaces after injection when it warms to body temperature and has a short half-life, delivering more than 90% of its therapeutic radiation within 10 days. Over time, natural breakdown products of RadioGel^®^ include lactic acid and glycolic acid (also known as non-toxic natural byproducts of the Krebs cycle), and the remaining radioactivity is excreted via urine [127].
- Hydroxyapatite (Ca_10_(PO_4_)_6_(OH)_2_) is a natural mineral constituent of bone matrix and, hence, is biocompatible. Hydroxyapatite particles can be easily synthesized in the desired particle-size range for embolization, and the abundant PO_4_^3−^ moieties can coordinate ^166^Ho. Earlier studies have investigated hydroxyapatite lanthanum oxide composites [128] and the effect of tissue engineering strategies on bone regeneration [129]. The synthesis of these particles requires heating in an oven at 1250 °C [130]. Hydroxyapatite particles were uniformly spherical and large (50 µm), with a high specific surface area, uniform mesopores, and a doxorubicin loading capacity of 460.8 µg mg^−1^. In vivo, hydroxyapatite particles could be smoothly delivered through an arterial catheter to achieve chemoembolization. Doxorubicin-loaded hydroxyapatite particles effectively inhibited liver cancer cell growth in a rabbit liver tumor model, demonstrating the efficacy of TACE [131]. Pre-clinical studies explored the possibility of using hydroxyapatite particles with a 20–60 μm size range for vascular occlusion [78,132]. After 6 weeks of therapy, the biodegradation of the hydroxyapatite particles was realized by metabolizing Ca^2+^ and PO_4_^3−^ ions [83,84].
- ^166^Ho-poly L-lactic acid (PLLA) microspheres have been developed as a possible alternative to TARE with glass- or resin-containing ^90^Y, as PLLA is biocompatible with the human body and its degradation reaction is mainly due to hydrolysis to lactic acid [75,77]. The chelated and stable form of Ho, Sm, or Y is added as an acetylacetonate compound and mixed with L-Lactic acid (LLA) polymer during microsphere polymerization [77,133]. When the particles are formed and isolated, they are irradiated with neutrons, which form the radioactive ^166^Ho, ^153^Sm, or ^90^Y [75].
- Macro-aggregate albumin particles from HSA-B20 (Rotop Pharmaka, Dresden, Germany), Vasculosis® (Global Medical Solutions, Auckland, New Zealand), MAA (DRAXIMAGE^®^, Kirkland, QC, Canada), Pulmocis® Curium (London, UK)) are prepared after heating albumin and can be labeled directly with ^99m^Tc, a recipe routinely used for scout scans in TARE set-up [99,103,134]. Alternatively, different therapeutic approaches have been investigated for TACE [49] and TARE [86], but, to date, only ^188^Re-labeled human serum albumin (^188^Re-HSA) microspheres have made their way to the clinic [85]. One advantage of HSA is that it is an approved carrier molecule, with ^99m^Tc-HSA (Vasculosis^®^, Nanocoll^®^ (GE Healthcare Ltd., Milan, Italy), Nanoalbumon^®^ (Radiopharmacy Laboratoy Ltd, Budaörs, Hungary), Magnevist^®^ (Bayer Inc., Toronto, ON, Canada) routinely used in nuclear medicine centers, indicated for blood pool imaging, angiocardiography, and ventriculography [135]. Pre-clinical [87] and clinical feasibility studies with ^188^Re-MAA have been published [65,85]. Both clinical studies demonstrated high product stability, low urinary excretion, good tolerance, and acceptable toxicity. Larger cohorts are necessary to conclude the usefulness of this device, which seems to be the ideal match with ^99m^Tc-MAA. More recently, ^90^Y-DTPA-HSA microspheres were successfully evaluated in rats [68]. In pre-clinical settings, MAA was functionalized with adamantane to allow a pre-targeting set-up in the liver of mice. With this, targeting and imaging with a radiolabeled CD-PIBMA-Cy5 polymer yielded uptake in the vasculature with the functionalized MAA in the liver of mice based on host–guest chemistry [136,137]. Recently, a similar pre-clinical setup was carried out using click chemistry based on the interaction between azide-functionalized MAA and a radiolabeled DBCO-carrying moiety [138]. Given these pre-clinical findings, forming HSA microspheres seems feasible and can be carried out at low costs in a GLP facility [85].
- Chitosan, a polymer of 2-deoxy-2-amino-d-glucose obtained from the exoskeletons of crustaceans such as crabs and shrimps, transforms from a liquid to a gel state above pH 6 [99]. Chitosan is also regularly used in nanoparticle vaccines [139]. The feasibility of chitosan for TAE was assessed in the renal arteries of a rabbit model [140]. The renal arteries were still completely occluded after 8 weeks, and no inflammatory reaction was observed. Several strategies are available to modify chitosan, which can be used to couple additional moieties of interest [141,142]; for example, pre-activation of the COOH-bearing label (e.g., adamantane) with DIC or EDC in (acidic) water can be followed by the addition of chitosan PyBOP base to subsequently conjugate the COOH-bearing label. Kim et al. studied doxorubicin-loaded chitosan microcapsules in TACE in rabbits [143]. In a recent study, biodegradable chitosan was used to deliver and retain ^166^Ho at the tumor site [31,79]. Chitosan was complexed with ^166^Ho after mixing ^166^HoCl_3_ or ^166^Ho(NO_3_)_3_ at pH 3 for 30 min [144,145]. The holmium/chitosan complex (Millican, Dong Wha Pharmaceutical Co., Seoul, Korea) was effective in treating small HCCs in a novel study based on 40 patients with single HCC < 3 cm in size with satisfactory response rates and survival rates of 87.2%, 71.8%, and 65.3% at 1, 2, and 3 years, respectively [79,89,90].
- Starch-based microparticles (SBMP) were proposed as a unique system for the pre-therapeutic step (scout scan) after ^188^Re or ^68^Ga radiolabeling and TARE after direct radiolabeling with ^188^Re using SnCl_2_ reduction with gluconate or with ^68^GaCl_3_ and sodium acetate [93]. SBMP appeared to be a promising theranostic agent for the internal radiation therapy of hepatocellular carcinoma. SBMP was first developed for lung perfusion scintigraphy and formulated as a ready-to-use ^99m^Tc radiolabeling kit [91,92]. After selecting suitable size particles via mechanical filtration, an aldehyde is formed, followed by the attachment of a diamine-linker. This chemistry should be possible with an amine, for example, Ahx [91,92,93]. The in vivo stability of the compounds is of primary importance, especially considering the therapeutic one, i.e., the SBMP radiolabeled with ^188^Re, and further investigations in pre-clinical models are warranted.

## 5. Advancements and Future Perspectives for Therapeutic Loads

TACE has been employed as drug-eluting microspheres, where embolic microspheres loaded with positively charged drugs release them locally to the tumor site via ion exchange, thus reducing systemic drug exposure [146,147]. The drug of choice for loading into microspheres is doxorubicin, a cytotoxic agent that interferes with DNA tumor growth [148]. Anti-angiogenic strategies are being considered in combination with drug-eluting particles to combat resistance [149]. It has been shown in pre-clinical and clinical settings that hypoxic conditions can lead to doxorubicin resistance in HCC cells and angiogenic upregulation of the formation of new blood vessels feeding the tumor cells [150,151]. 

Regarding TARE, most of the innovations lie in further integrating scout scans and actual therapy delivery. One strategy that has been suggested is the use of low-dose therapeutic spheres for scout scans [152]. While ensuring that the particles behave identically, this strategy potentially puts the patient at risk. Alternatives have also been sought after. Recent pre-clinical studies report on the possibility of microsphere surface modifications in combination with host–guest [136,137,153,154] and “click” chemistry [155] strategies. In these pre-targeting approaches, after functionalization with either adamantane (guest-vector) or azide moieties, microspheres of aggregated albumin (MAA) create a platform for the introduction of therapeutic moieties. This feature further integrates the scout scan with the therapeutic delivery. Uniquely, this strategy allows for the exploratory use of cells as “bio-microspheres” [156].

Recently, developments have been made in the direction of targeted α-therapy (TAT), using α-emitting isotopes such as ^225^Ac [157], ^211^At, and ^223^Ra [158]. Generally, α-emitting isotopes are effective in tissue penetration up to less than 0.1 mm, whereas with β-emitting isotopes, the radiation range in tissue varies between 2 and 12 mm, depending on their β-energies. Due to their shorter range, higher linear energy transfer, and presence of therapeutic daughter nuclides, α-particles are ideal for local tumor treatment [159]. For embolization therapy with interest in the platform of TARE, advances can be made using a-emitting radioisotopes instead of b-emitting radioisotopes. A point to consider is the radiochemistry to prepare a-emitting isotopes inside microspheres.

The BCLC system (Barcelona Clinic Liver Cancer) is recommended as a staging and treatment algorithm for HCC. For patients who are not a candidate for curative treatments, locoregional therapies, as discussed in this review, are recommended. On the other hand, the BCLC system does not recommend systemic molecular therapy for early-stage HCC. The choice of treatment depends on the availability of the discussed treatment options, besides patients’ costs and toxicity. Where TARE and TACE can be considered, this will partly depend on the patient’s characteristics, tumor size and number, local availability and expertise, and, of course, the decisions made during the tumor board meeting. The meta-analysis of Chow et al. showed a similar overall survival (OS) for the use of RFA, TACE, and TARE. Further research is in a more homogeneous group [160].

## 6. Conclusions

A range of materials has been used for TAE, TACE, and TARE. Hereby, the chemical composition of the microspheres guided the presence of a therapeutic load, indicating that specific healthcare needs require specific chemical designs.

## Figures and Tables

**Table 1:** Properties of microspheres: list of requirements for embolization (selected from: https://www.microspheres.us/properties-of-microspheres/ accessed on 1 November 2022).

Property	Importance
Specific gravity (particle density)	Dispersion in other media or occlusion of the micro-vasculature
Size	Particle size (diameter = 50–750 mm) that allows occlusion of the microvasculature
Durability	Strength during production, solvent resistance, sterilization, chemical stability, or biodegradation, the release of the therapeutical payload
Biocompatibility	Safety, toxicity, stability, suitable for intra-arterial delivery
Pharmacology	Controlled dosimetry and dosing, full control over release profile by diffusion, zero-order kinetics
Surface properties	Hydrophobic vs. hydrophilic surface, surface area, and porosity, ability to coat or functionalize the spheres

**Table 2:** Overview of non-functionalized microspheres for hepatic trans-arterial embolization (TAE). PC = pre-clinical use, H = Human use, FDA = FDA approved as an active implantable medical device. See Section 4 for a detailed evaluation of the microspheres.

Microspheres Composition	Product Name	Particle Size Range (⌀ μm)	PC/H (FDA Clearance)	Biodegradable	References
Tris acryl gelatin microspheres (TAGM)	Embosphere^®^ (Merit Medical Systems, South Jordan, UT, USA)	100–300, 300–500	H (FDA)	No	[24]
Polyvinyl alcohol (PVA) sodium acrylate co-polymer microspheres	Contour SE^®^ (Boston Scientific, Marlborough, MA, USA), Bead Block^®^ (Boston Scientific, Marlborough, MA, USA) Embozene^®^ (Varian Medical Systems, Palo Alto, CA, USA)	Irregular: 150–250, 250–355, 355–500, 500–710	H (FDA)	No	[25]
Polyvinyl alcohol-based hydrogel microspheres with sulphonate groups	LC Bead^®^ (Boston Scientific, Marlborough, MA, USA)	75–150, 100–300, 300–500, 500–700	H (FDA)	No	[26,27,28,29]
Co-polymer of PEG and diacrylamide	Hydropearl^®^ (Terumo Medical Co., Somerset, NJ, USA)	75–1100	H (FDA)	No	[1]
Starch microspheres	Embocept^®^ (Pharmacept, Berlin, Germany), Spherex^®^ (Magle Life Sciences, Lund, Sweden)	50	PC	Yes	[1]
Gelatin microspheres	Gel-Bead (Teleflex, Morrisville, NC, USA)	100–300, 300–500, 500–700, 7000–1000	H (FDA)	Yes	[1]
Collagen-coated poly-(DL-lactic acid-co-glycolic acid (PLGA) microspheres	Occlusin500^®^ (IMBiotechnologies, Edmonton, AL, Canada)	150–210	H	Yes	[1]

**Table 3:** Drug-eluting microspheres for functionalized hepatic TACE. PC = pre-clinical use, H = human use, FDA = FDA approved as an active implantable medical device. See Section 4 for a detailed evaluation of the microspheres.

Microspheres Composition	Product Name	Particle Size Range (⌀ µm)	Drug Load	PC/H (FDA)	Biodegradable	References
Polyvinyl alcohol (PVA) sodium acrylate co-polymer microspheres	QuadraSphere^®^ and HepaSphere™ (Merit Medical Systems, Inc., South Jordan, UT, USA) DC Bead^®^ (Boston Scientific, Marlborough, MA, USA), LC Bead^®^, and Bead Block^®^ (Boston Scientific, Marlborough, MA, USA)	50–100, 100–300, 200–400	Doxorubicin, irinotecan, epirubicin, oxaliplatin	PC/H (FDA)	No	[14,26,32,33,34,35,36]
Ion-exchange microspheres	CalliSpheres^®^ Beads (Jiangsu Hengrui Medicine Co. Ltd. Jiangsu, China)	100–300	Doxorubicin, pirarubicin, oxaliplatin	PC/H	No	[37,38,39,40,41]
Tris acryl gelatin microspheres (TAGM)	Embosphere (Merit Medical Systems, South Jor-dan, UT, USA), Embozene^®^, and Oncozene™ (Varian Medical Systems, Palo Alto, CA, USA)	40–120, 100–300	Doxorubicin and Irinotecan	H (FDA)	No	[13,36,42,43]
Poly-lactide-co-glycolide (PLGA)	Dexon^®^, Vicryl^®^, Perseris^TM^, Indivior (Indivior Inc. North Chesterfiled, VI, USA), Risperdal Consta^®^	20–100	Mitomycin, doxorubicin, irinotecan, sunitinib, cisplatin	PC/H (FDA)	Yes	[44,45,46,47]
Albumin microspheres	Nab-paclitaxel	10–220	Mitomycin C, doxorubicin, paclitaxel	PC/H (FDA)	Yes	[48,49]

**Table 4:** Microspheres used for trans-arterial radioembolization (TARE). PC = pre-clinical use, H = human use, FDA = FDA approved as an active implantable medical device. See Section 4 for a detailed evaluation of the microspheres.

Microspheres Composition	Product Name	Particle Size Range (⌀ µm)	Radioisotope Load	Pre-Clinical/ Human Use (FDA Clearance)	Biodegradable	References
Glass	Lipiocis, TheraSphere^®^ (Boston Scientific, Marlborough, MA, USA)	50–150, 20–30, 25–32	^32^P, ^90^Y, ^177^lu, ^186^Re, ^188^Re	PC/H (FDA for ^90^Y, ^186^Re, and ^188^Re)	No	[60,61,62,63,64,65]
Resin	SIR-Spheres^®^ (Sirtex Medical Inc. Woburn, MA, USA), Amberlite IR-120 (Thermo Fisher Scientific, Landau, Germany)	20–60	^90^Y, ^153^Sm	PC/H (FDA for ^90^Y)	No	[66,67,68,69]
Polyhydroxyamic acid polyacrylamide (PHA)	Experimental	54	^177^lu, ^131^I	PC	No	[70,71,72]
Styrene divinylbenzene	Amberlite IR-120 (Thermo Fisher Scientific, Landau, Germany)	20–40	^152^Sm	PC	No	[69,73]
Poly- DL-lactic acid-co-glycolic acid (PLGA)	YPO_4_ crystalline particles Radiogel^®^ (Vivos Inc., Richland, WA, USA)	0.5–2	^90^Y	PC (FDA-approved as a medical device)	Yes	[74]
Poly (L-lactic acid) PLLA	Resomer^®^ L104 (Merck, Darmstad, Germany)	10–45, 20–40	^188^Re/^166^Ho/^175^Yb	PC/C	Yes	[75,76,77,78,79,80,81]
Poly (glycidyl methacrylate-*co*-ethylene dimethacrylate & Quinoline-8-ol	G-Gel (Merck, Darmstad, Germany)	20–40	^131^I, ^177^lu	PC	No	[66,70,71,72,82]
Hydroxyapatite	QuiremSpheres (Quirem Medical, Deventer, The Netherlands)	20–60	^166^Ho	PC	No	[78,83,84]
Albumin	HSA-B20 (Rotop Pharmaka, Dresden, Germany) Vasculosis^®^ (Global Medical Solutions, Auckland, New Zealand) MAA (DRAXIMAGE^®^, Kirkland, QC, Canada), Pulmocis^®^ (Curium, London, UK)	25–35, 15–37	^90^Y, ^186^Re, ^188^Re	PC/C	Yes	[63,64,65,85,86,87]
Chitosan	Millican (Dong Wha Pharmaceutical Co., Soeul, South Korea)	5–20	^166^Ho	PC/C	Yes	[75,76,77,78,79,88,89,90]
Starch-based microparticles (SBMP)	Experimental Kit	18–42	^188^Re	PC	No	[91,92,93]

## Data Availability

The data are available upon request from the corresponding author.
